# Risk of Cardiovascular Events Associated with Inhaled Corticosteroid Treatment in Patients with Chronic Obstructive Pulmonary Disease: A Meta-Analysis

**DOI:** 10.1155/2018/7097540

**Published:** 2018-07-15

**Authors:** Xia Jing, Yufeng Li, Jianying Xu

**Affiliations:** ^1^Department of Respiratory Diseases, Shanxi Dayi Hospital Affiliated to Shanxi Medical University, Taiyuan, Shanxi, China; ^2^Department of Neurology, Shanxi Dayi Hospital Affiliated to Shanxi Medical University, Taiyuan, Shanxi, China

## Abstract

**Background:**

The cardiovascular (CV) safety of inhaled corticosteroids (ICSs) in chronic obstructive pulmonary disease (COPD) is controversial because different studies have suggested that ICSs either increase or reduce the risk of CV events in COPD patients. In this meta-analysis, we assess the CV safety of ICS therapy in COPD.

**Methods:**

A meta-analysis of randomized, double-blind, parallel-group, placebo-controlled trials of ICS treatment for COPD that include at least 4 weeks of follow-up was performed. A random-effects model was used to evaluate the effects of ICS treatment on CV events. CV events were documented in each trial, and the relative risk (RR) and 95% confidence intervals (CIs) for ICSs were estimated.

**Results:**

Thirty-one trials were included in this meta-analysis. The risk of CV events was not different between ICS-treated and control groups (RR: 0.99; 95% CI: 0.93 to 1.06; *P*=0.801). In a subgroup analysis, there were no significant differences in CV events between an ICS combined with long-acting *β*_2_ agonist (LABA) (ICS + LABA) group and an LABA-only group (RR: 1.00; 95% CI: 0.90 to 1.10; *P*=0.930), as well as between a combination group (ICS + LABA) and a long-acting muscarinic antagonist (LAMA) combined with LABA (LAMA + LABA) group (RR: 0.78; 95% CI: 0.39 to 1.55; *P*=0.473). In addition, there was no difference in the risk of CV events between ICS treatment and control groups (RR: 0.99; 95% CI: 0.90 to 1.09; *P*=0.872).

**Conclusions:**

These results demonstrate that ICSs do not increase the risk of CV events in COPD patients.

## 1. Introduction

Chronic obstructive pulmonary disease (COPD) is currently the fourth leading cause of death in the world [1] and is projected to be the third leading cause of death worldwide by 2020 [2]. Inflammatory changes in the airway and lung parenchyma are responsible for pulmonary function decline [3]. Inhaled corticosteroids (ICSs) are effective in reducing airway inflammation, nonspecific airway hyperresponsiveness, and airway obstruction in patients with COPD [4]. An ICS combined with a long-acting *β*_2_ agonist (LABA) is more effective than monotherapy in improving lung function, reducing symptoms, increasing exercise tolerance, decreasing the frequency of exacerbation and hospitalization, and improving patient health [5]. According to the current Global Initiative for Chronic Obstructive Lung Disease (GOLD) guidelines, ICSs in combination with LABAs are suggested for patients with moderate-to-very severe COPD [6]. ICSs, such as fluticasone and budesonide, are widely used in COPD treatment, and other formulations of ICSs approved for use in COPD are fluticasone with salmeterol and budesonide with formoterol [7].

COPD is significantly associated with subclinical atherosclerosis [8], a recognized marker of cardiovascular (CV) disease. CV disease, which is a frequent and important comorbidity in COPD, is a fatal cause of morbidity and mortality among COPD patients during exacerbations [9]. ICSs may potentially reduce CV events by reducing exacerbations, alleviating hypoxia, and relieving the systemic inflammatory reaction to atherogenesis in patients with COPD [10]. Nevertheless, after prolonged treatment, the potential benefits of an ICS may be offset by its adverse systemic effects, such as increased extracellular volume, hypertension, glucose intolerance, and dyslipidemia, which are well-recognized risk factors for CV disease [11, 12]. Several studies have shown that ICS treatment can alleviate CV symptoms in COPD patients [3, 13–15], while other studies have suggested that ICSs lead to an increased risk of CV events [16, 17]. Critical evaluation is required to assess whether ICS treatment can reduce or increase the risk of CV events in COPD patients. Therefore, we conducted this meta-analysis to evaluate the effect of ICS treatment on CV events in COPD patients compared with controls.

## 2. Materials and Methods

### 2.1. Register and Search Strategy

This systematic review and meta-analysis were reported in accordance with the Preferred Reporting Items for Systematic Reviews and Meta-Analyses (PRISMA) Statement and registered at the International Prospective Register of Systematic Reviews (number CRD42017066017).

We searched PubMed, Embase, the Cochrane Library, Web of Knowledge, the United States Food and Drug Administration (US FDA) website, the http://ClinicalTrials.gov database, and the GSK (GlaxoSmithKline) Clinical Study Register for eligible trials. The search strategy used for PubMed was “inhaled corticosteroids” OR “ICS” OR “budesonide” OR “fluticasone” OR “flunisolide” OR “beclomethasone” OR “beclometasone” OR “triamcinolone” AND “COPD” OR “chronic obstructive pulmonary disease” OR “chronic obstructive lung disease” OR “COAD” OR “chronic obstructive airway disease” OR “pulmonary disease, chronic obstructive” OR “airflow obstruction, chronic” OR “chronic airflow obstruction” AND “mortality” OR “death” OR “myocardial” OR “cardiovascular” AND “clinical trial,” based on previously published meta-analyses [18, 19]. We also conducted a manual search using the reference lists of key articles published in English. We considered all potentially eligible studies for review, irrespective of the primary outcome or language. Those searches aimed at identifying studies which were published between January 1, 1980, and January 24, 2018.

### 2.2. Eligibility Criteria

The inclusion criteria for our trials were as follows: (1) studies that were randomized controlled trials (RCTs) with a double-blind, parallel-group, placebo-controlled design and more than 4 weeks of follow-up and the title or abstract including any ICS (flunisolide, fluticasone, beclomethasone, budesonide, or triamcinolone); (2) studies that used standardized diagnostic criteria for COPD (spirometry is required for diagnosis in this clinical context, and the presence of a postbronchodilator FEV1/FVC (forced expiratory volume in the first second/forced vital capacity) ratio <0.70 confirms the presence of a persistent airflow limitation, and thus the presence of COPD, in patients with appropriate symptoms and significant exposures to noxious stimuli [20]); (3) studies that included any subtype of COPD, severity of disease, and sex or race of study participant subjects; (4) studies that used an ICS as the intervention drug versus a control treatment, which consisted of an ICS versus placebo, an ICS in combination with an LABA versus an LABA alone, or the combination of an ICS and an LABA versus the combination of a long-acting muscarinic antagonist (LAMA) and an LABA; (5) trials that provided the details of CV events (coronary artery disorders, cardiac arrhythmias, heart failures, cardiac disorder signs and symptoms, myocardial disorders, cardiac valve disorders, pericardial disorders, central nervous system vascular disorders, arteriosclerosis, stenosis, vascular insufficiencies and necrosis, aneurysms and artery dissections, embolisms, thrombosis, and hypertension) and explicitly reported data (including zero events) for at least one such event, but the outcome does not include stroke or peripheral arterial disease. New onset cases of cardiac disorders and cardiovascular death were included; and (6) studies whose full text can be found on a search site.

### 2.3. Exclusion Criteria

Our exclusion criteria were as follows: (1) studies that were less than 4 weeks in duration, as we were interested in long-term CV event risks; (2) studies that mixed groups of participants with asthma; (3) trials that did not compare an ICS with another treatment; and (4) studies that provided no raw data regarding the number of CV events in ICS-treated patients with COPD.

### 2.4. Outcome Measures and Data Extraction

The initial outcomes were CV adverse events. The CV events were defined as coronary artery disorders, cardiac arrhythmias, heart failures, cardiac disorder signs and symptoms, myocardial disorders, cardiac valve disorders, pericardial disorders, central nervous system vascular disorders, hypertension, arteriosclerosis, stenosis, vascular insufficiencies and necrosis, aneurysms and artery dissections, embolisms, and thrombosis. Two reviewers (XJ and YFL) independently and individually extracted the outcome data with an agreement value (*κ*) of 94.5%, and a third reviewer (JYX) provided additional insight when a discrepancy occurred. If the primary outcomes were not available in the original article, we searched for details using http://ClinicalTrials.gov, the US FDA website, and the manufacturer's clinical trial registry website. We used the Jadad scoring system to evaluate all trials [21], and a score >2 was required for a trial to be kept in our analysis.

### 2.5. Statistical Analysis

We pooled trial data using Stata Version 12.0 and calculated the relative risk (RR) and 95% confidence intervals (CIs) for the primary outcomes. A two-sided *α* value of 0.05 was defined as statistically significant. The magnitude of heterogeneity was estimated by the *I*^2^ statistic and Cochran's *Q* test, and an *I*^2^ value greater than 50% was indicative of moderate-to-high heterogeneity. If substantial statistical heterogeneity was found, we explored the sources of heterogeneity and the effect of individual study characteristics and subgroups on the risk estimates. The funnel plots and Begg's and Egger's tests were used to assess the publication bias.

Subgroup analyses were conducted with the data separated by three different groups (ICS versus placebo, ICS + LABA versus LABA, and ICS + LABA versus LAMA + LABA). An influence analysis was performed to evaluate the influence of individual studies on the summary effect. Because the effect of ICS on CV disease is a long-term effect, we conducted a sensitive analysis including studies with longer-term follow-up (>2 years).

## 3. Results

### 3.1. Characteristics of the Identified Studies

**Figure 1:**
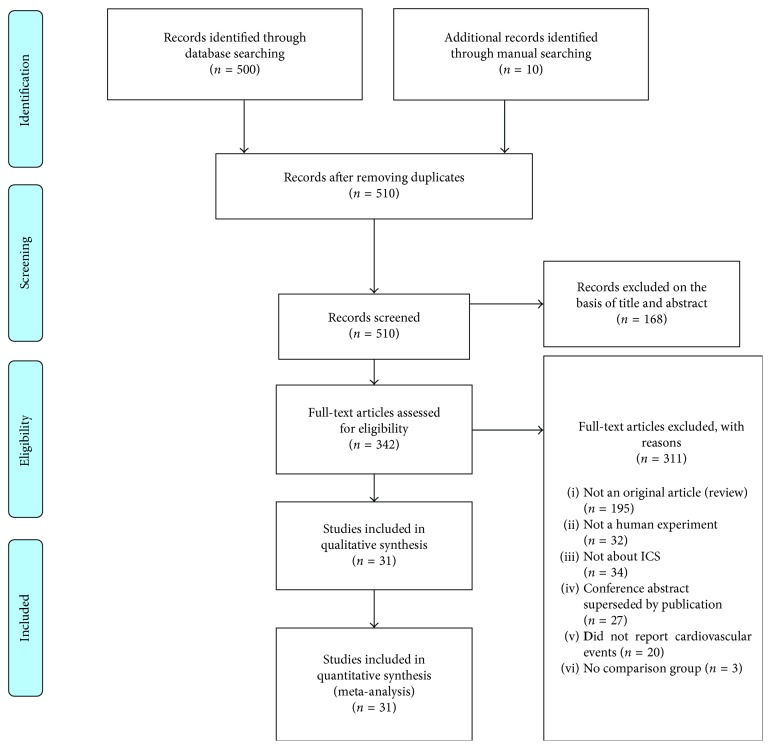
Study selection process.

A total of 31 trials including 57031 patients fulfilled the inclusion criteria and were selected for analysis. The details of the study selection strategy are shown in Figure 1, and the main characteristics of the included trials are shown in Table 1. All trials were of high quality (Jadad score >2). These trials enrolled a total of 29171 participants who received an ICS and 27860 participants who received control therapies. Among the 31 studies, the main outcome of 13 studies was cardiovascular events, but there was no exact definition of cardiovascular events in these studies. In 31 studies, 5 studies resulted in cardiac arrhythmias, 2 in atrial fibrillation, 3 in cardiac failure, 3 in cardiac ischemia, 1 in acute myocardial infarction, 1 in angina pectoris, 1 in coronary artery stenosis, 1 in acute coronary syndrome, 2 in hypertension, and 15 in cardiovascular death. Inhaled fluticasone was evaluated in 26 trials [3, 16, 17, 22–44], inhaled budesonide in 4 trials [15, 45–47], and inhaled beclomethasone in only 1 trial [48]. The range of mean age in patients was from 52.4 to 67.6. The majority of the participants were male, with the proportion of current smokers ranging from 24% [22] to 83% [31]. The duration of the trials ranged from 4 to 156 weeks, with 18 trials being longer than 52 weeks in duration [3, 15, 22–26, 28, 30, 32, 33, 35, 39, 42, 44, 45, 47]. Most trials enrolled participants with severe COPD, as the mean predicted FEV1 of the participants was >50% in 7 trials [15, 23, 29, 33, 37, 38, 47] compared to ≤50% in 17 trials [3, 16, 17, 22, 24, 28, 30–32, 34, 36, 45, 46, 48], whereas 7 trials did not report the details of FEV1.

The modified Jadad scale is used to assess the methodological quality of a clinical trial judging the effectiveness of blinding. The score range is from zero (very poor) to seven (rigorous).

### 3.2. Main Findings

**Figure 2:**
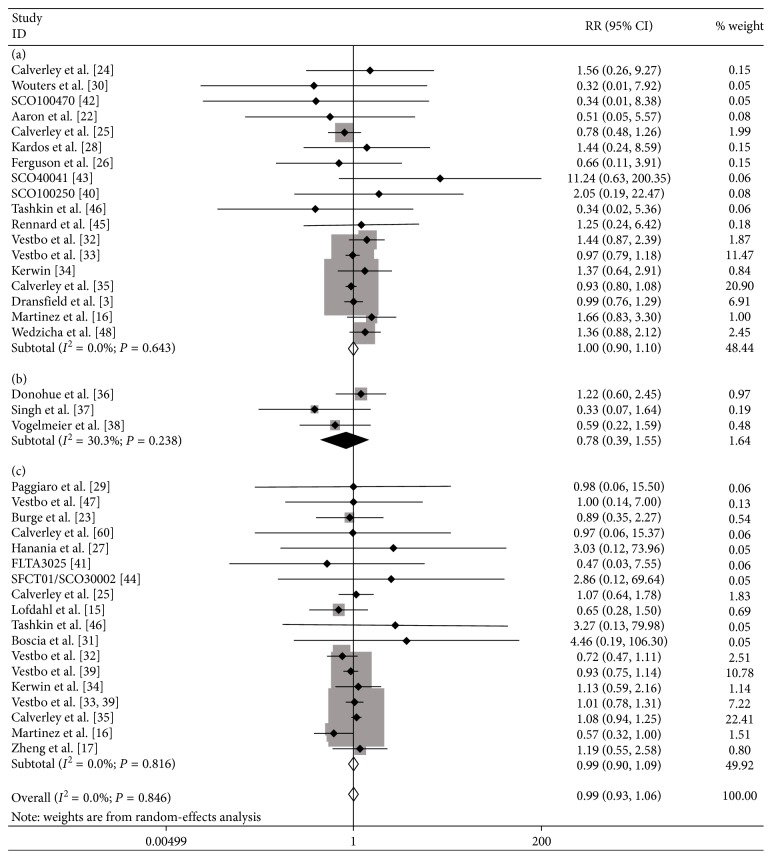
Meta-analysis of RCTs of ICSs versus controls for risk of CV events. (a) ICS + LABA versus LABA; (b) ICS + LABA versus LAMA + LABA; (c) ICS versus placebo. LABA: long-acting *β*_2_ agonist; LAMA: long-acting muscarinic agonist.

In total, all 31 studies reported at least one CV event. The ICS treatment did not increase the risk of CV events in COPD patients compared with the controls (RR: 0.99; 95% CI: 0.93 to 1.06; *P*=0.801; Figure 2), and there was no evidence of statistical heterogeneity (*I*^2^ = 0%, *P*=0.846).

### 3.3. Subgroup Analyses

ICS use was not associated with a significant effect on the risk of CV events when used in the combination of ICS + LABA compared to LABA alone (RR: 1.00; 95% CI: 0.90 to 1.10; *P*=0.930 (Figure 2); *I*^2^ = 0%, *P*=0.643). In addition, there was no significant difference between the ICS + LABA combination and the LAMA + LABA combination in the number of CV events (RR: 0.78; 95% CI: 0.39 to 1.55; *P*=0.473 (Figure 2); *I*^2^ = 0%, *P*=0.238). ICS treatment did not increase the risk of CV events compared with placebo (RR: 0.99; 95% CI: 0.90 to 1.09; *P*=0.872 (Figure 2); *I*^2^ = 0%, *P*=0.816).

### 3.4. Sensitivity Analysis

We implemented a sensitivity analysis by using influence analysis to verify whether an individual study influenced the results, and no combined outcomes were changed by any given study. In analysis including studies with a longer-term period (>2 years), the pooled RR and 95% CI were 0.99 (0.92–1.06), and there was no evidence of statistical heterogeneity either (*I*^2^ = 0%, *P*=0.787).

### 3.5. Publication Bias

**Figure 3:**
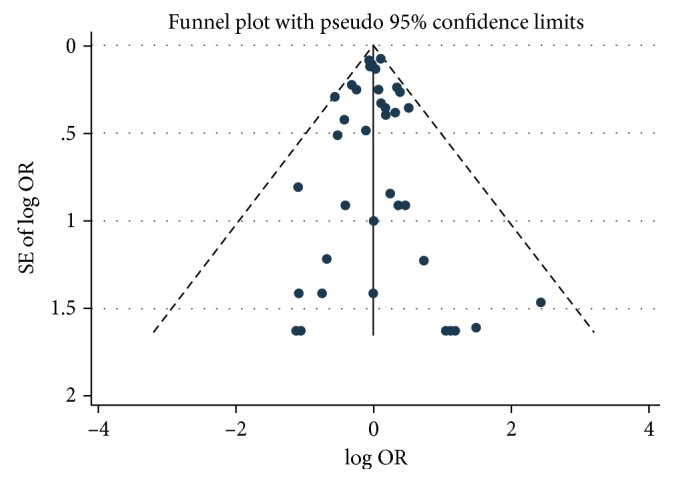
Funnel plots of RCTs of ICSs versus controls for risk of CV events.

Moreover, the funnel plot that was used to assess publication bias appeared to be symmetrical and showed no publication bias (Figure 3). Meanwhile, there was no publication bias shown using Egger's test (*P*=0.733). In sensitivity analysis, there was no publication bias shown using Egger's test either (*P*=0.735).

## 4. Discussion

Our meta-analysis did not show any consistent association between ICS therapy and CV events in COPD patients, either as monotherapy or in combination with LABAs, when compared to placebo, LABA, and LABA/LAMA controls.

COPD is not simply a pulmonary disease [49]; it is associated with increased extrapulmonary complications. COPD often coexists with other chronic diseases that can influence patients' physical status and prognosis [50]. Patients with COPD are at higher risk of CV events than age-matched and sex-matched individuals without COPD [9, 51]. Meanwhile, more patients with COPD die from CV events than from the respiratory consequences of airflow limitation [52] because the hypoxemia, respiratory alkalosis caused by hyperventilation, and inflammation in people with lung function decline could result in higher CV morbidity and mortality [53]. In contrast, prevention and treatment of exacerbations have been identified by GOLD as a priority since they are associated with impaired pulmonary function. Consequently, treatments that increase lung function and reduce exacerbations [54] would be expected to reduce both respiratory and adverse CV events. The recommendation of ICS therapy combined with an LABA in patients with moderate-to-very severe airflow limitations is based on evidence for reduced exacerbations. The pathophysiology of COPD includes systemic inflammation disorder and/or alterations in repair mechanisms. There are potential mechanisms linking COPD with an increased risk of CV events, including common risk factors (e.g., smoking), systemic inflammation [55], and vascular dysfunction [56]. The overexpression of inflammatory mediators into the circulatory system of COPD patients is relevant to the progression of CV events [57]. A mechanism for the reduction in CV-related deaths associated with ICS therapy is not clear. Potential explanations include a reduction in COPD exacerbations, which lead to hypoxia and instability that may predispose patients to CV events, a reduction in systemic inflammatory reactions to atherosclerosis [58], or a reduction in adaptive immune responses. Nevertheless, ICS therapy may have limited CV benefits because ICS treatment does not reduce the inflammatory marker levels in systemic circulation (serum levels of C-reactive protein or interleukin 6), and ICS therapy also has little impact on neutrophil inflammatory responses or extracellular matrix remodeling linked to CV disease in COPD [57, 59].

The results of our analysis are consistent with other published studies showing that ICSs have no increased risks of CV events in COPD patients. Calverley et al. [60] reported that most of the deaths were events related to COPD, and only a few were related to CV events in COPD patients treated with budesonide. Furthermore, Calverley et al. [35] observed that the probability of COPD patients having an adverse CV event within 3 years of treatment was 24.3% for fluticasone propionate (FP) and 24.2% for placebo. Moreover, Dransfield et al. [3] reported that different doses of fluticasone furoate/vilanterol (FF/VI) combinations did not increase the risk of CV events compared with the risk associated with vilanterol (VI) alone. In addition, Loke et al. [61] concluded that ICS use was not associated with a significant risk of myocardial infarction when combined ICS + LABA therapy was compared to LABA treatment alone (RR: 0.92; 95% CI: 0.63 to 1.35; *P*=0.67) or when ICS treatment was compared to placebo (RR: 0.97; 95% CI: 0.67 to 1.40; *P*=0.87). They also found that ICS use was not associated with a significant risk of CV-related death when combined ICS + LABA therapy was compared to LABA treatment alone (RR: 1.12; 95% CI: 0.79 to 1.58; *P*=0.53) or when ICS was evaluated against placebo (RR: 0.95; 95% CI: 0.71 to 1.27; *P*=0.74). According to a recent study, the CV endpoint in the fluticasone furoate (FF) (hazard ratio (HR): 0.90; 95% CI: 0.73 to 1.1) groups did not differ from that in the placebo group [33]. Meanwhile, FF/VI had no effect on composite CV events (HR: 0.93; 95% CI: 0.75 to 1.14) with similar findings for VI (HR: 0.99; 95% CI: 0.80 to 1.22) [33].

There are certain discrepancies between the results of our meta-analysis and the outcomes of previous studies. A number of studies have suggested that ICSs may potentially confer CV benefits. Two observational studies [13, 14] reported a significant association between ICS exposure and a reduction in CV-related deaths but did not specify details on the causes of death. Macie et al. [14] concluded that an ICS-induced reduction in mortality was particularly notable for CV-associated deaths but not for COPD. Lee et al. [13] found that ICS exposure was associated with a 20% decrease in the odds of a CV-related death (odds ratio (OR): 0.80; 95% CI: 0.72 to 0.88). A pooled analysis of these two studies showed a significant reduction in CV-associated deaths (RR: 0.79; 95% CI: 0.72 to 0.86; *P* < 0.0001) [61]. Similar beneficial effects were also verified in RCTs. Lofdahl et al. [15] found that patients treated with inhaled budesonide had a significantly lower incidence of ischemic cardiac events (RR: 0.58; 95% CI: 0.35 to 0.98; *P*=0.043) than those receiving placebo. Additionally, in a previous study [3], the risk of CV events decreased with increasing doses of FF; specifically, there was a CV benefit of FF/VI therapy compared with VI therapy alone with respect to patient-reported outcomes. Nevertheless, ICSs were not associated with a significantly reduced risk of myocardial infarction (RR: 0.83; 95% CI: 0.63 to 1.08) in an observational study [62]. Furthermore, another analysis conducted by Calverley et al. [25] showed that there was no significant reduction in cardiac disorders among patients treated with fluticasone (reported as CV event rates per study year: 0.113 in the placebo group, 0.102 in the fluticasone group, 0.114 in the salmeterol group, and 0.087 in the combination therapy group). Conversely, another study [16] suggested that FF/VI treatment resulted in increased CV effects compared with VI treatment alone. Similarly, in a study of Asian patients treated with FF/VI, high-dose FF was associated with increased CV events compared to those with low-dose FF [17].

There are several limitations to our research that make it difficult to reach a definitive conclusion. First, the RCTs did not use specific definitions of a CV event, and inconsistent adverse event reporting may result in some missing outcome data. Second, most of the included trials were not specifically designed to monitor the risk of CV events, which might have resulted in incomplete reporting of CV events. Third, the data used in this analysis were based on the judgment of the investigators, which might result in discrepancies between studies. Despite these limitations, we believe that our analysis adds more positive evidence for CV safety of ICS therapies in COPD patients. We will investigate the intraclass differences in the risk of CV events between different ICS therapies in our future meta-analysis. Our follow-up reanalysis of patient-level data (gender, age, smoking history, pulmonary function, and preexisting ICS use) may clarify the optimal role of ICS use in COPD.

## 5. Conclusions

In summary, despite certain limitations, our findings still have potential implications. We performed a meta-analysis of 31 RCTs, and the outcome indicates that ICSs do not increase the risk of CV events in COPD patients. This analysis also provides further evidence of safety for this important treatment option. Further studies are needed to validate our results in independent cohorts of patients with larger sample sizes and a wider range of clinical courses.

## Figures and Tables

**Table 1:** Characteristics of included studies.

Author	Year	J	Drug	Male (%)	Mean age (years)	FEV1 (% predicted)	Current smokers (%)	Treatment duration (weeks)
Aaron et al. [22]	2007	7	SFC versus SAL	57.9	67.5 ± 8.9	39.4 ± 11.9	32.4	52
				57.4	67.6 ± 8.2	38.0 ± 13.1	24.3	
Burge et al. [23]	2000	5	FP versus placebo	75	63.7 ± 7.1	50.3 ± 14.9	36.4	156
				74.2	63.8 ± 7.1	50.0 ± 14.9	39.2	
Calverley et al. [24]	2003	5	SFC versus SAL; FP versus placebo	75	62.7 ± 8.7	44.8 ± 14.7	52	52
				70	63.2 ± 8.6	44.3 ± 13.8	51	
				70	63.5 ± 8.5	45 ± 13.6	53	
				75	63.4 ± 8.6	44.2 ± 13.7	47	
Calverley et al. [25]	2007	6	SFC versus SAL; FP versus placebo	75	65 ± 8.3	44.3 ± 12.3	43	156
				76	65.1 ± 8.2	43.6 ± 12.6	43	
				75	65 ± 8.4	44.1 ± 12.3	43	
				76	65 ± 8.2	44.1 ± 12.3	43	
Ferguson et al. [26]	2008	7	SFC versus SAL	58.3	64.9 ± 9.0	39.8 ± 13.9	40	52
				52.0	65.0 ± 9.1	50.6 ± 15.4	38	
FLTA3025 [41]	2000	5	FP versus placebo	66	63.3 ± 10	NA	NA	24
				72	65.2 ± 8.7			
Hanania et al. [27]	2003	7	SFC versus SAL	61	63 ± NA	41 ± 11	43	24
				58	64 ± NA	42 ± 12	51	
Kardos et al. [28]	2007	4	SFC versus SAL	74	63.8 ± 8.3	40.4 ± 8.9	49.7	52
				77.6	64 ± 8.2	40.3 ± 8.5	49.9	
Lofdahl et al. [15]	2007	5	Budesonide versus placebo	73.5	52.5 ± 7.5	76.8 ± 12.4	39.4	156
				72.2	52.4 ± 7.7	76.9 ± 13.2	39.2	
Paggiaro et al. [29]	1998	5	FP versus placebo	99	62 ± NA	59 ± 18	49	24
				78	64 ± NA	55 ± 17	49	
Rennard et al. [45]	2009	6	Budesonide/FOR versus placebo	62.5	63.2 ± 8.9	33.8 ± 11.4	34.8	52
				65.3	62.9 ± 9.1	35.5 ± 11.9	39.5	
SCO100250 [40]	2007	6	SFC versus SAL	51	65.4 ± NA	NA	NA	52
				57	65.3 ± NA			
SCO100470 [42]	2005	6	SFC versus SAL	78.3	63.5 ± 9.3	NA	42	24
				77.2	63.7 ± 9.0		44	
SCO40041 [43]	2007	5	SFC versus SAL	59.7	65.4 ± 8.4	NA	NA	156
				62.7	65.9 ± 9.5			
SFCT01/SCO30002 [44]	2005	5	FP versus placebo	83.9	64.6 ± 8.7	NA	NA	52
				80	65.7 ± 9.0			
Tashkin et al. [46]	2008	4	Budesonide/FOR versus FOR	67.9	63 ± NA	33.7 ± 11.8	40.8	26
				65.5	64 ± NA	33.6 ± 11.3	38.4	
			Budesonide versus placebo	67.6	63 ± NA	33.5 ± 10.8	40.0	
				69.0	63 ± NA	34.6 ± 10.5	36.0	
Vestbo et al. [47]	1999	5	Budesonide versus placebo	58.6	59.0 ± 8.3	86.2 ± 20.6	75.9	156
				62.1	59.1 ± 9.7	86.9 ± 21.1	77.2	
Wouters et al. [30]	2005	4	SFC versus SAL	73	63 ± 7.9	47.4 ± 13.9	39	52
				75	64 ± 7.7	48.2 ± 12.9	35	
Boscia et al. [31]	2012	4	FF/VI versus placebo	46	57.9 ± 9.2	49.8 ± 10.6	83	4
Vestbo et al. [32]	2009	4	FP versus placebo	75	64.8 ± NA	44.7 ± NA	43	144
				77	64.9 ± NA	44.4 ± NA	42	
			SFC versus SAL	75	64.9 ± NA	44.8 ± NA	43	
				77	64.8 ± NA	44.1 ± NA	43	
Vestbo et al. [39]	2016	6	FF versus placebo	74	65 ± 8	59.6 ± 6.1	47	152
				75	65 ± 8	59.7 ± 6.1	47	
			FF/VI versus VI	76	65 ± 8	59.7 ± 6.1	45	
				75	65 ± 8	59.7 ± 6.1	47	
Kerwin et al. [34]	2013	4	FF versus placebo	64	62.7 ± 9.5	41.5 ± 13.13	54	24
				68	62.1 ± 8.8	42.4 ± 12.80	54	
			FF/VI versus VI	67	62.3 ± 8.5	42.3 ± 12.74	54	
				68	63.4 ± 9.6	44.5 ± 12.78	54	
Dransfield et al. [3]	2013	6	FF/VI versus VI	57.3	63.6 ± 9.1	45.7 ± 12.9	NA	52
				58.4	63.6 ± 9.4	44.3 ± 13.2		
Calverley et al. [35]	2010	7	SFC versus SAL	75	65.0 ± 8.3	NA	43	144
				76	65.2 ± 8.2		43	
			FP versus placebo	75	65.1 ± 8.4		43	
				76	65.1 ± 8.1		43	
Martinez et al. [16]	2013	5	FF/VI versus VI	71	61.9 ± 8.8	48.1 ± 12.85	53	24
				74	61.2 ± 8.6	48.5 ± 12.89	55	
			FF versus placebo	74	61.8 ± 8.3	48.4 ± 12.17	56	
				74	61.9 ± 8.1	48.3 ± 12.71	53	
Donohue et al. [36]	2015	5	FSC versus UMEC/VI	69	63.0 ± 8.91	48.3 ± 10.82	41	12
				72	62.5 ± 9.05	48.6 ± 10.71	45	
Singh et al. [37]	2015	4	FSC versus UMEC/VI	71	61.4 ± 8.06	51.1 ± 10.50	61	12
				73	61.8 ± 7.94	50.2 ± 10.85	57	
Zheng et al. [17]	2015	5	FF/VI versus placebo	93	65.1 ± 9.19	49.6 ± 13.19	52	24
				90	64.7 ± 8.78	48.6 ± 13.39	56	
Wedzicha et al. [48]	2014	5	BDP/FOR versus FOR	69	64.6 ± 8.6	41.9 ± 6.0	39	48
				69	63.9 ± 8.6	41.6 ± 6.0	40	
Vogelmeier et al. [38]	2013	7	SFC versus QVA149	71.6	63.4 ± 7.7	60.0 ± 10.7	48.1	48
				70.2	63.2 ± 8.2	60.5 ± 10.5	47.7	
Vestbo et al. [33]	2016	7	FF/VI versus placebo	50	67 ± 10	NA	45	144
				48	67 ± 10		47	

The quality (Q) of each study was based on the Jadad scoring system. J: Jadad score; FP: fluticasone propionate; SAL: salmeterol xinafoate; SFC: combination of salmeterol xinafoate and fluticasone propionate; BDP: beclomethasone dipropionate; FOR: formoterol; TIO: tiotropium; UMEC: umeclidinium; QVA149: combination of indacaterol (a long-acting *β*_2_ agonist) with glycopyrronium (a long-acting muscarinic antagonist) as a dual bronchodilator; FF: fluticasone furoate; VI: vilanterol; NA: not applicable.
